# M1 and M2 Macrophages Differentially Regulate Colonic Crypt Renewal

**DOI:** 10.1093/ibd/izad270

**Published:** 2023-11-24

**Authors:** Sathuwarman Raveenthiraraj, Griselda Awanis, Marcello Chieppa, Amy E O’Connell, Anastasia Sobolewski

**Affiliations:** School of Pharmacy, University of East Anglia, Norwich Research Park, Norwich, Norfolk, NR4 7TJ, UK; Division of Newborn Medicine, Boston Children’s Hospital, Boston, MA, 02115, USA; School of Pharmacy, University of East Anglia, Norwich Research Park, Norwich, Norfolk, NR4 7TJ, UK; Department of Biological and Environmental Sciences and Technologies, University of Solento, Centro Ecotekne, 73043, Monteroni, Lecce, Italy; Division of Newborn Medicine, Boston Children’s Hospital, Boston, MA, 02115, USA; Department of Pediatrics, Harvard Medical School, Boston, MA, 02115, USA; School of Pharmacy, University of East Anglia, Norwich Research Park, Norwich, Norfolk, NR4 7TJ, UK

**Keywords:** stem cells, Lgr5, macrophages, colon, inflammation

## Abstract

**Background:**

The colonic epithelium is the most rapidly renewing tissue in the body and is organized into a single cell layer of invaginations called crypts. Crypt renewal occurs through Lgr5 + gut stem cells situated at the crypt base, which divide, produce daughter cells that proliferate, migrate, differentiate into all the cells required for normal gut function, and are finally shed into the crypt lumen. In health, this rapid renewal helps maintain barrier function next to the hostile gut microbial luminal environment. Inflammation results in an influx of immune cells including inflammatory M1 macrophages into the gut mucosa next to the crypt epithelium, but the direct effect of macrophages on crypt regeneration and renewal are poorly understood.

**Methods:**

Using an in vitro macrophage-crypt coculture model, we show that homeostatic M2 macrophages and inflammatory M1 macrophages confer different effects on the crypt epithelium.

**Results:**

Both M1 and M2 increase crypt cell proliferation, with M2 macrophages requiring physical contact with the crypt epithelium, whereas M1 macrophages exert their effect through a secreted factor. Only M1 macrophages reduce goblet and Tuft cell numbers and increase Lgr5 + crypt stem cell numbers, all dependent on physical contact with the crypt epithelium. Further studies showed that M1 macrophages increase the Wnt signaling pathways cyclin D1 and LEF1 through physical contact rather than a secreted factor.

**Conclusions:**

These findings highlight the importance of understanding distinct cellular interactions and direct dialogue between cells and increase our understanding of the contribution of different immune cell subtypes on crypt cell biology during inflammation.

**Key Messages**

**What is already known?**

Tolerogenic intestinal macrophages are abundantly present in the microenvironment of the colonic epithelium and its stem cell niche, while aberrant infiltration of inflammatory macrophages contributes to intestinal disease pathogenesis.

**What is new here?**

Inflammatory (M1) macrophages regulate colonic epithelial cell differentiation through direct macrophage-crypt contact, not observed with anti-inflammatory (M2) macrophages. Crypt cell growth is induced by both macrophage subsets, but for M1, it occurs via a secreted factor, while M2 requires physical contact with the crypt epithelium.

**How can this study help patient care?**

Understanding the physical interactions of gut epithelial cells and macrophages in health vs inflammation will help identify new therapeutic targets for inflammatory bowel disease that regulate epithelial cell growth and differentiation.

## Introduction

The intestinal epithelium, lining the innermost layer of the large intestine, plays a crucial role in the physical protection of the underlying tissue from pathogenic threats, commonly encountered in the lumen, where a single-cell thick epithelium is perpetually renewed every 4 to 5 days.^1^ Leucine-rich repeat containing G protein-coupled receptor 5 (Lgr5) expressing stem cells at the base of epithelial invaginations, termed crypts, drive epithelial renewal.^2^ Here, Lgr5+ intestinal stem cells in the colon generate highly proliferative transit amplifying daughter cells, which migrate along the crypt-axis and give rise to fully differentiated epithelial cells such as enterocytes, goblet cells, tuft cells, and enteroendocrine cells until they are shed into the lumen at the end of their life cycle.^3^

To further counteract the looming threat the large microbial presence poses over the colonic epithelium, the underlying lamina propria employs the densest macrophage population in the human body, where blood derived Ly6C+ monocytes are recruited to the submucosa where they then differentiate towards a mature macrophage phenotype.^4^ Through their proximal peri-cryptal localization in the lamina propria, these macrophages swiftly apprehend invasive foreign pathogens in a tolerogenic manner, while an escalating inflammatory response is repressed.^5-9^ Macrophages are highly plastic, and their phenotypical properties are often influenced through environmental cues within the lamina propria.^10^

Early studies have broadly defined 2 distinctive macrophage phenotypes based on their physiology and function commonly known as M1 and M2 macrophages.^11^ Here, acute epithelial injury results in the influx of pro-inflammatory and bactericidal subsets of M1 macrophages, while residential macrophages in the steady-state reportedly possess an M2-like macrophage phenotype.^12,13^ Interestingly, the transcriptional profile of macrophages present in patients with Crohn’s disease (CD) and ulcerative colitis (UC) closely aligns with the definition of the M1 macrophage phenotype and is postulated to contribute to disease progression.^14^

Over the last decade, gene signature studies have postulated that the M1 and M2 activation states likely represent the opposite ends of the phenotypical macrophage spectrum.^11^ Here, several studies have demonstrated that M1 macrophages express distinct pro-inflammatory cytokines and chemokines compared with its M2 macrophage counterpart, where the cytokine profile is dominated by the expression of anti-inflammatory associated chemokines such as IL-10 and TGF-β among other.^15^^,^^16^ Furthermore, it has been established that M1 and M2 macrophages can be defined by their relative expression of CD38, where M1 are mostly CD38^+^.^17^

The classical role of macrophages in tissue clearance and intestinal immunity has been extensively studied, however, as M1 and M2 macrophages often cohabitate the submucosal space in vivo, little is known regarding their respective capacity to engage with the colonic epithelium and their respective contributory role in epithelial renewal.^5^

Indeed, ablation of the macrophage population in the small intestine resulted in the marked reduction of Lgr5 + expressing stem cells and reduced intestinal motility.^18,19^ Furthermore, early work from Pull et al, demonstrated that a subset of activated macrophages are recruited to the site of injury and induce proliferation of epithelial progenitor cells within the crypt, while Skoczek and colleagues further showed that inflammatory monocytes, a macrophage precursor, are recruited and juxtaposed to Lgr5EGFP + stem cells at the base of colonic crypts upon exposure to *E.coli* in vivo and induced an increase in epithelial proliferation in vitro.^20,21^

As cell-to-cell contact between two cell types may evoke a signaling cascade in the target cell, collectively these studies suggest that macrophages likely function as a secondary mediator of the intestinal stem cell niche. However, it is unclear whether secretory factors or physical contact is utilized to regulate the intestinal stem cell niche. Regardless, it begs the question of whether the phenotypic states of M1 and M2 macrophages that are commonly exhibited during intestinal inflammation and steady state, respectively, can differentially regulate colonic crypt renewal.

The intestinal lamina propria plays an essential role in the maintenance of the colonic stem cell niche, where the underlying mesenchymal, immune cells, or extracellular matrix compartments were demonstrated to modulate the stem cell niche.^22–24^ However, due to the myriad of subepithelial signaling stimuli involved, in vivo models face challenges in delineating their respective effects on the stem cell niche. As most adult intestinal macrophages are derived from the monocytic cell lineage, we are able to mirror the in vivo crypt-macrophage microenvironment using our in vitro reductionist 3D coculture model, allowing for the close spatial-temporal study of bone marrow–derived M1 and M2 macrophage interactions and its effects on colonic crypt renewal.^21^

We show that both M1 and M2 macrophage can increase colonic crypt proliferation, while M1 macrophages can induce colonic crypt proliferation through secreted factors. We further demonstrate that juxtracrine contact between M1 but not M2 macrophages results in decreased tuft and goblet cell expression, while observing an increase in Lgr5-expressing stem cell numbers, where direct M1 macrophage-epithelial interactions result in the upregulation in downstream Wnt (Wingless/Integrated)-signaling targets LEF1 (Lymphoid enhancer binding factor 1) and CyclinD1 (G1/S-specific cyclin-D1) in the colonic epithelium.

## Materials and Methods

### Mouse Studies

All animal experiments were conducted in accordance with the Home Office Animals (Scientific procedures) Act of 1986, with approval of the University of East Anglia Ethical Review Committee, Norwich, United Kingdom. Female C57BL/6 (UEA-Disease Modelling Unit) aged between 8 and 12 weeks were euthanized by CO_2_ asphyxiation and subsequent cervical dislocation in accordance with Schedule 1 of the Act.

### Isolation and Culture of Bone Marrow–Derived Macrophages

Following the isolation of the femur/tibia and the removal of residual connective tissue, the bone’s epiphyses were severed, and the bone marrow was flushed in a sterile environment using a 28-gauge syringe and cold RPMI (Roswell Park Memorial Institute) 1640 (+10% FBS, +1% Pen/Strep, Gibco). The flushed bone marrow contents were then then filtered through a 70-μm nylon cell strainer (Falcon) and collected in a 50-mL centrifuge tube (Falcon). Following centrifugation at 600 *g* for 10 minutes, the cell suspension was resuspended in warm RPMI1640. A bone-marrow yield was established, and the cells were seeded onto 6-well ultra-low attachment plates (Corning) at a cell density of 1 × 10^6^ cells/mL. To drive BMDM (bone-marrow derived macrophages) differentiation towards macrophages, supplementary murine RM-CSF (Peprotech) at a concentration of 0.2 μg/mL was added on day 0 and 3 and macrophages were harvested on day 8.

### Polarization of Macrophage Population

Macrophages were polarized based on methods previously described by Ying et al in 2013. The BMDM cells were cultured in RPMI 1640 media up to day 7. On day 7, the floating cell population was removed, and the media was replaced by new fresh media. For M1 activation, supplementary LPS (100 ng/mL) and interferon (IFN)- γ (50 ng/mL) were added to the media for a further 24 hours; and for M2 activation, interleukin (IL)-4 (10 ng/mL) and IL-13 (10 ng/mL) were added instead.

### Isolation and Culture of Murine Colonic Crypts

Colonic crypts were isolated from the distal colon of C57BL/6 mice, as previously described by Skoczek and colleagues.^21^ Briefly, following the culling of the mouse, the colon was removed and washed with ice-cold PBS (Phosphate buffered saline) to remove excess fecal matter; the colon was then cut longitudinally, and excess mucus within the tissue was gently dissociated. Next, 0.5-mm tissue pieces were placed in a saline solution [50 mL dH_2_O with NaCl [140 mM], KCl [5 mM], HEPES [10 mM], d-glucose [5.5 mM], Na_2_HPO_4_ [1 mM], MgCl [0.5 mM], CaCl [1 mM], EDTA [1 mM], DTT [0.153 μg/mL], L-glutamine [200 mM], Pen/Strep [200 U/mL] and NEAA [2%]) for 1 hour. To liberate the crypts, the solution containing the tissue was shaken to aid gentle dissociation and then collected following crypt sedimentation. The single crypts were embedded in growth factor-reduced matrix Matrigel (VWR) and seeded onto No.0 glass coverslips (0.08-0.13 mm) contained within 12-well plates (Starlab). Following polymerization of the Matrigel after 8 minutes at 37°C, the coverslips were flooded with colonic crypt culture media (advanced DMEM/F12, containing B27 [20 μl/mL], N2 [10 μl/mL], N-acetyl-L-cysteine [0.163 μg/mL], HEPES [10 mM], Pencillin/Streptomycin [100 U/mL], GlutaMAX [2 mM], EGF [50 ng/mL], Noggin [100 ng/mL; all from Peprotech], Wnt-3A [200 ng/mL] and R-spondin-1 [1 mg/mL, BioTechne]).

### Coculture of Macrophages and Colonic Crypts

To isolate the macrophage population, cells were harvested on day 8. On day 8, the adherent population was liberated using 0.48 mM Versene. The optimum macrophage seeding density was previously determined to be 5.7 × 10^5^ cells per well, which was then added to the colonic crypt/Matrigel solution. The mixture was then seeded onto a No.0 glass coverslip (Thermofisher). Following Matrigel polymerization at 37°C, the Matrigel was then flooded with colonic crypt culture medium (as described previously).

### Culture of Colonic Crypts with Macrophage-Conditioned Media

Macrophages and crypts were isolated and cultured as previously described previously. To study macrophage secretory factor-derived effects on colonic crypts, four conditioned-media culture models were devised. Under the “control crypt” model, 2 separated Matrigels with colonic crypts alone are seeded onto a well. Under the “M1 coculture” model, 2 separated Matrigels seeded with M1 macrophages and colonic crypts were seeded onto a well. Under the M1 conditioned media (CM) model, 2 separated Matrigels with M1 macrophages seeded along with colonic crypts, and another seeded with crypts alone was cultured. Under the “M1 only” model, 2 separated Matrigels, one with colonic crypts seeded alone and another with M1 macrophage seeded alone, were cultured onto a well. For EdU (5-ethynyl-2'-deoxyuridine) incorporation experiments, the “control crypt,” “M1/M2-crypt coculture,” and “M1/M2 conditioned media” setup was utilized. The diagram in Supplementary Figure 3 summarizes the experimental setup described previously.

### Immunofluorescent Labelling

For characterizing cells within the coculture system, epithelial-specific antibodies were used. Following the coculture, the coverslips were fixed with 4% PFA (paraformaldehyde) for 1 hour on ice. Washing steps were carried following each step. Ammonium chloride (100 mM in PBS, pH 7.4) was added to each coverslip for 13 minutes, washed with PBS, followed by further incubation with 10% SDS (sodium dodecyl sulfate) in PBS (pH 7.4) for 5 minutes. Next, 1% Triton-X was added for 30 minutes to permeabilize the organoids. Nonspecific binding was inhibited using 10% donkey or goat serum (Gibco, depending on antigen retrieval) for 20 minutes.

Primary antibodies for enteroendocrine cells (CgA+, Abcam), tuft cells (DCAMKL1+, Abcam), Caspase 3 (cell signaling), or stem cells (Lgr5+, Origene) were added for overnight incubation at 4°C. The following day, immunolabelling was visualized using a species-specific Alexa-Fluor-conjugated secondary antibodies (488, 568, 647) raised in mouse, donkey, goat, or rabbit and added for 2 hours at 4°C. PE (Phycoerythrin)-conjugated Ulex europaeus lectin (UEA-1) was acquired from VectorLabs to label goblet cells. Finally, the slides were washed and mounted with Hoechst/Vectashield (VectorLabs); the slides were later visualized using an epifluorescence or confocal microscopy.

### Colonic Crypt EdU Incorporation Experiments

Colonic crypts were cultured as previously described. After 24 hours, EdU (10 µM) was added and incubated at 37°C/5% CO_2_ overnight. On day 2, the crypts were fixed and processed as described previously and EdU incorporation detected through a Click-iT reaction as per manufactures’ instructions (Thermofisher).

### Image Analysis

All fluorescent images were captured on the equatorial plane of the crypt as previously described^25^ using either a Nikon TI with a x20 0.4 NA, Zeiss Axiovert 200 with a x20 NA objective or using a Zeiss LSM-510-META confocal microscope with a x63 1.4NA 0.75 mm WD oil immersion objective.

All images were analyzed with Fiji (Image J) software. To identify enteroendocrine cells (CgA+; Abcam), tuft cells (DCAMKL1+; Abcam), goblet cells (UEA-1+; Vectorlabs), Caspase 3 (Cell signaling) and stem cells (Lgr5+; Origene), Z-stacks were taken at a 1-μm intervals for 5 μm above and below the crypt equatorial plane, to ensure counting of cells only in the equatorial plane. For cell counting of crypt differentiation markers, the crypt was divided 3 crypt regions: base (cells within the +4 position of the crypt), mid, and top region. To identify the stem cells within a crypt, the basal Lgr5 expression of each cell across the Z- stack (optical slices) 5 μm above and below the equatorial plane was counted. Crypt budding numbers were quantified by counting the buds present on day 1 and day 6 of culture.

### Quantification of Nuclear Fluorescence Intensity

Images were captured using the confocal microscope (LSM-510-META) with a x63 1.4NA 0.75 mm WD oil immersion objective. To quantify the expression of Cyclin-D1 and LEF1 within the nucleus, the average fluorescence value of every nucleus present at the equatorial plane was measured. Using Fiji Image J’s polygon tool, the nuclear area was identified by following the perimeter of each individual DAPI+ nuclei in the equatorial plane. The arbitrary fluorescent value of the channels occupied by Cyclin-D1 and LEF1 were then measured as shown in Supplementary Figure 4.

### Statistical Analysis

All experiments were repeated at least 3 times unless stated otherwise. Data are expressed as mean ± standard error of mean (SEM, *n* = number of independent experiments, N = minimum total number of crypts), and a minimum of 20 crypts per experiment were counted. Statistical analysis was carried out using the Graphpad Prism 9 software. Comparisons between 2 or more groups were measured using 1-way ANOVA with post hoc Tukey analysis, and a paired *t* test was utilized to compare differences between 2 groups. A *P* value of less than 0.05 was considered statistically significant.

## Results

### M1 and M2 Macrophages Stimulate the Proliferation of Colonic Crypts

**Figure 1.**
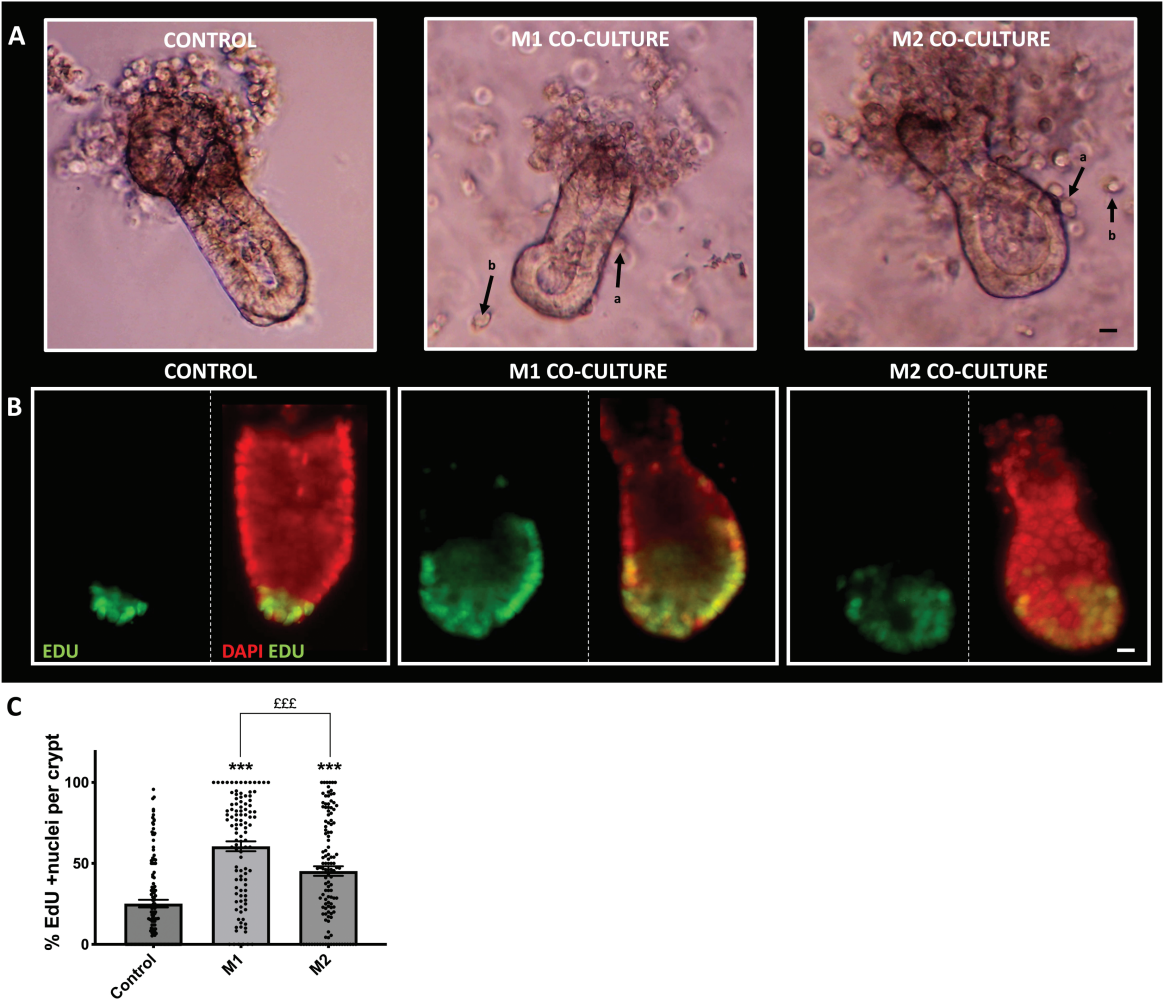
The M1 and M2 macrophages increase EdU incorporation of colonic crypts in in vitro coculture. A, Representative white light images showing crypts cultured alone and with M1 or M2 macrophages, where macrophages are either (a) in contact or (b) not in contact with crypts (white arrows). Scale bar at 15 µm (B) Representative epi-fluorescent images showing EdU incorporation (green) in the nuclei (red) within colonic crypt-macrophage cocultures. Colabelling of nucleus and EdU shown in yellow (C) Histogram showing the percentage of EdU positive nuclei per crypt within the macrophage subtype coculture condition. (n = 3, ***P < .001 compared with control; M1 compared with M2 £££ *P* < .001). Scale bar at 15 µm.

To determine the effects M1 and M2 macrophages have on colonic crypt growth, we cultured M1 or M2 macrophages along with freshly isolated crypts, where macrophages are either in contact or proximally localized near the crypts (Figure 1A). Primarily macrophages were found to be in contact with the base and mid region of the crypt (Supplementary Figure 2). We then examined EdU incorporation in the colonic epithelium using immunofluorescent microscopy (Figure 1B). The coculture of crypts with either M1 or M2 macrophages resulted in a significant increase in EdU incorporation (green) compared with control (Figure 1B). Most notably, EdU incorporation was also significantly higher in crypts cultured with M1 macrophages when compared with M2 macrophages. Further analysis of epithelial caspase-3 expression (Supplementary Figure 1) did not show any significant changes between crypts cultured with M1 or M2 macrophages compared with control, while morphological analysis of colonic crypt length signified a shortening of crypt length in crypts cultured with M1 macrophages compared with control crypts (Supplementary Figure 2C**).**

### M1 Macrophages But Not M2 Macrophages Reduce Goblet and Tuft Cell Numbers in Colonic Crypts

**Figure 2.**
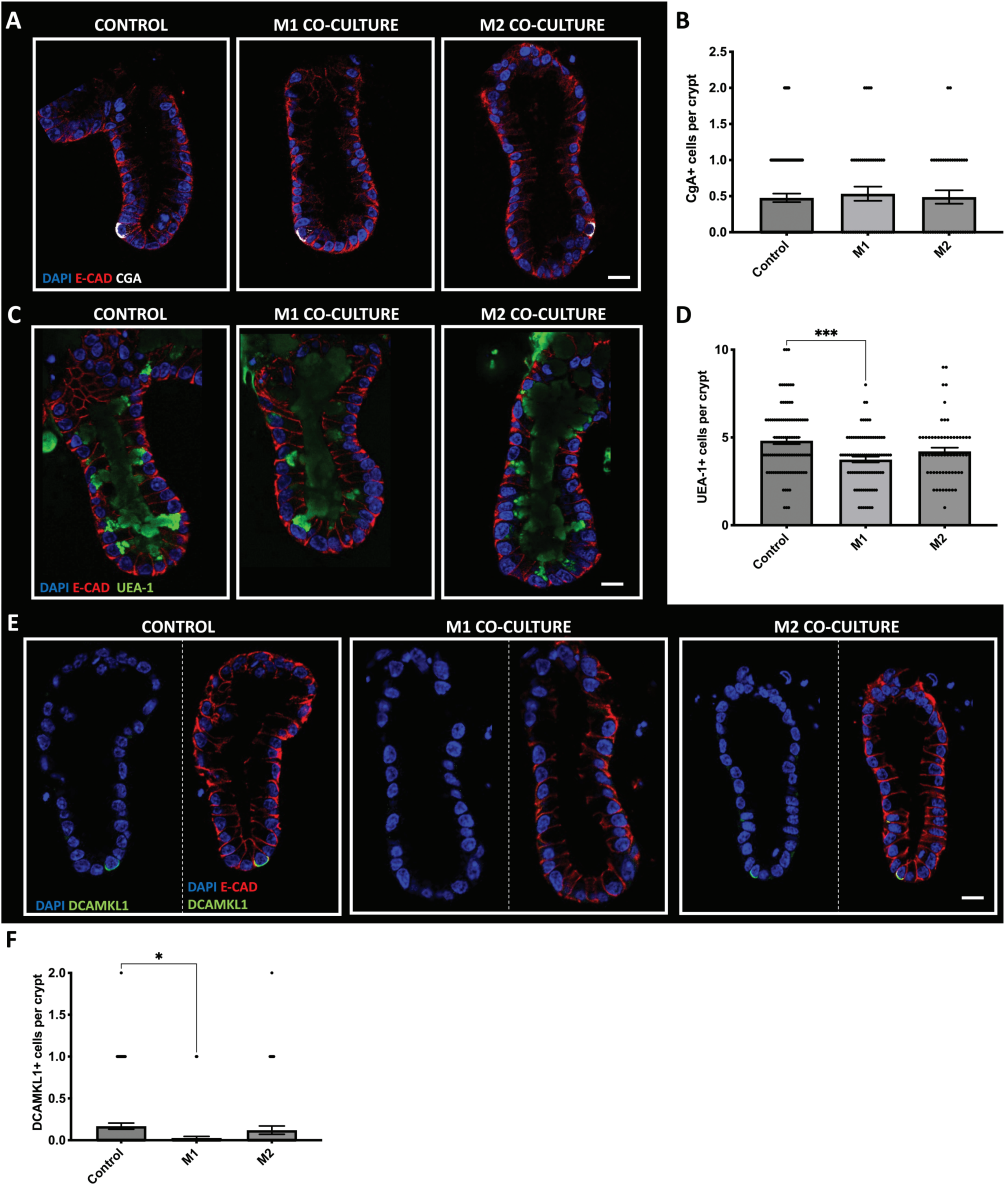
CgA + cell numbers are maintained in crypts cultured with M1 or M2 macrophages, while UEA-1 + goblet cells and DCAMKL1 + tuft cell numbers are decreased in crypts cultured with M1 macrophages but not M2. A, Representative confocal images showing Chromogranin-A(CgA) expression (white), DAPI (blue) and E- cadherin (red) in crypt-macrophage subtype coculture. B, Histogram showing the average number of CgA positive cells per crypt within each coculture condition (*n* = 4, ns). C, Representative confocal images showing UEA-1 expression (green), DAPI (blue) and E-cadherin (red) in each crypt-macrophage subtype coculture. D, Histogram showing the average number of UEA-1 positive cells per crypt within each coculture condition (*n* = 5, **P < .01 ***P < .0001 compared with control). E, Representative confocal images showing DCAMKl1 expression (green), DAPI (blue) and E-cadherin (red) in each crypt-macrophage subtype coculture. F) Histogram showing the average number of DCAMKL1 positive cells per crypt within each coculture condition (*n* = 5, *P < .05 compared with control). Scale bar at 20 μm.

To determine whether M1 or M2 macrophages affect the differentiated cell compartment within colonic crypts, we aimed to quantify enteroendocrine, tuft, and goblet cell numbers using confocal microscopy (Figure 2).

Using chromogranin-A (white) and E-cadherin (red), we visualized CgA+ enteroendocrine cells present in crypts cultured with M1 or M2 macrophages compared with control (Figure 2A). Here we show that the culture of M1 or M2 macrophage with crypts does not significantly affect enteroendocrine cell numbers (Figure 2B).

Next, we used the Ulex europaeus agglutinin (UEA-1, green) and E-cadherin (red) to identify goblet cells within crypts cultured with M1 and M2 macrophages compared with control (Figure 2C). We show that the coculture of M1 macrophages with colonic crypts results in a significant decrease in UEA-1+ goblet cell numbers compared with control, while crypts cultured with M2 macrophage maintained UEA-1+ goblet cell numbers (Figure 2D).

To determine whether the tuft cell numbers were affected in crypts cultured with M1 or M2 macrophages, we visualized the epithelial tuft cell population using DCAMKL1 (green) and E-cadherin (red; Figure 2E). Here, we observed a significant reduction in DCAMKL1 + tuft cell numbers in crypts cultured with M1 macrophages compared with control, while DCAMKL1 + tuft cells were maintained in crypts cultured with M2 macrophages (Figure 2F).

**Figure 3.**
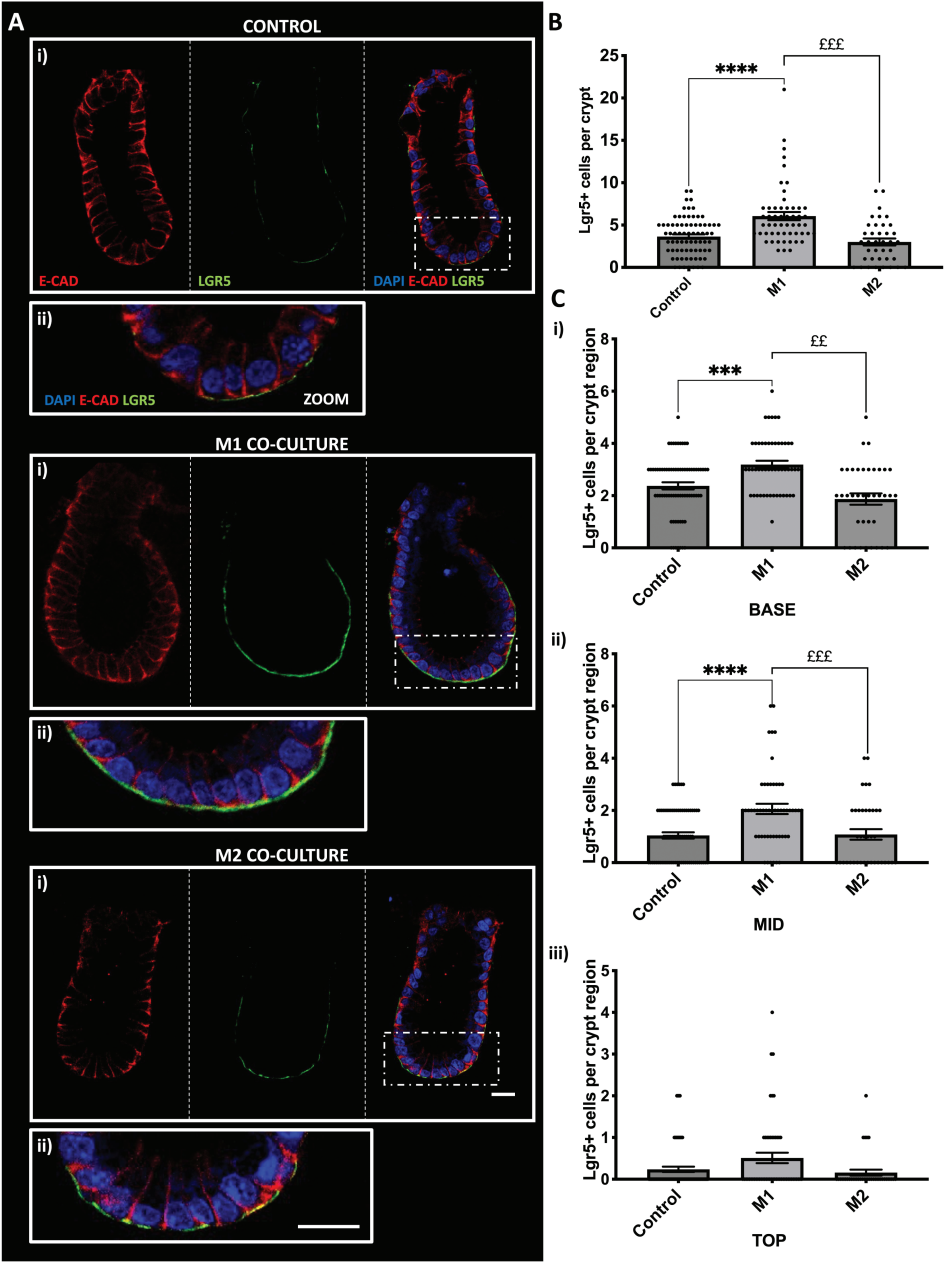
M1 but not M2 macrophages increase in vitro Lgr5+ cell expression in colonic crypts within the coculture model. A, (i) Representative confocal images showing Lgr5 expression (green), DAPI (blue), E-cadherin (red) and brightfield (white) in each crypt-macrophage subtype coculture and (ii) enlarged image of crypt base. B, Histogram showing the average number of LGR5 positive cells per crypt within each coculture condition. C, Histogram showing the position of Lgr5 positive cells within each crypt region (i) base, (ii) mid, and (iii) top (*n* = 4, **P < .01 compared with control, ***P < .001; M2 compared with M1 £££ *P* < .001; ££ *P* < .01). Scale bar 20 μm.

Having shown the effect of M1 and M2 macrophages on the differentiated crypt epithelial cell population, we wanted to further understand their effect on the epithelial stem cell population. Here we identified the colonic stem cell population using the leucine-rich G-protein coupled receptor 5, Lgr5 (green) and E-cadherin (red; Figure 3A). We observed a significant increase in Lgr5+ stem cell numbers in crypts cultured with M1 macrophages compared with control, while Lgr5+ stem cell numbers were comparable to control in crypts cultured with M2 macrophages (Figure 3B). Further analysis of Lgr5+ stem cell position within the colonic crypt compartment has shown that an increased number of Lgr5+ stem cells were localized in the base and mid region of crypts cultured with M1 macrophages when compared with control and crypts cocultured with M2 macrophages. No significant changes were noted in the top region of colonic crypts cultured with either M1 or M2 macrophages compared with control (Figure 3C).

### Epithelial Proliferation Is Increased via Secretory Factors in M1 Macrophages—M2 Macrophages Require Juxtracrine-Contact

**Figure 4.**
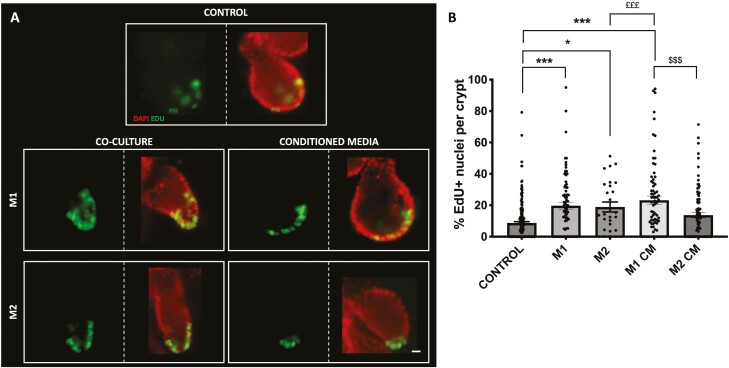
Juxtracrine contact is utilized by both M1 and M2 macrophages to induce increased EdU incorporation in colonic crypts, while M1 macrophages can further increase EdU incorporation via secretory factors in vitro. A, Representative epi-fluorescent images showing EdU incorporation (green) in the nuclei (red) within colonic crypt-macrophage coculture and conditioned media (CM). B, Histogram showing the percentage of EdU positive nuclei per crypt within each coculture and conditioned media (CM) culture model. (*n* = 4, compared with control *P < .05, **P < .01, ***P < .001; M1 compared with M2 $$*P* < .01). Scale bar at 15 μm.

After demonstrating that EdU incorporation significantly increased in crypts cultured with M1 and M2 macrophages, we next determined whether the previously observed effects derive from physical cell-cell contact between macrophages and colonic crypt cells as observed in vitro (Supplementary Figure 2B) or coculture-derived secretory products using a conditioned media model (Supplementary Figure 3A). Here we show representative images of EdU (green) incorporation in crypts cells (red) cultured in the presence of M1 or M2 macrophages (M1/M2 coculture) and crypts cultured without direct contact to M1 or M2 macrophages (M1/M2-CM; Figure 4A). As expected from previous results in Figure 1, M1 or M2 coculture crypts induced a significant increase in EdU incorporation when compared with control crypts. However, when crypts were cultured without direct contact with M1 macrophages (M1-CM), the crypt EdU incorporation was significantly increased compared with control crypts (Figure 4B). No significant changes in the percentage of EdU crypt incorporation was observed without direct contact with M2 macrophages (M2-CM) compared with control crypts.

### M1 Macrophage-Epithelial Juxtracrine Contact Is Required to Reduce Goblet and Tuft Cell Numbers

**Figure 5.**
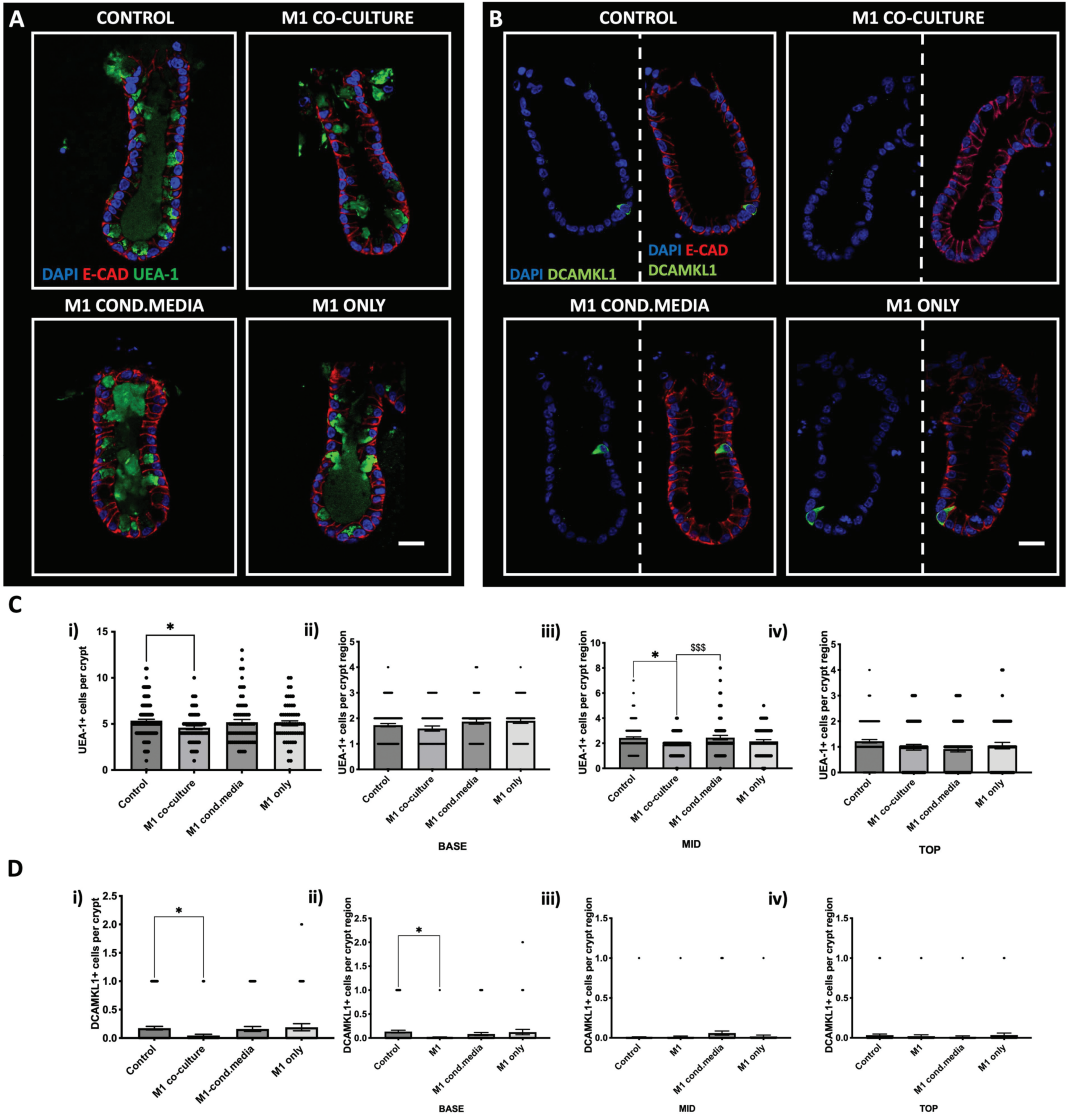
Physical contact between M1 macrophages and colonic crypts but not secretory factors decrease UEA-1+ and DCAMKL1+ cell expression in crypts in vitro. A, Representative confocal images showing UEA-1 expression (green), nuclei (blue) and E-cadherin (red) in crypts cultured in the M1 coculture, M-CM media and M1 only culture models. B, Representative confocal images showing DCAMKL1 expression (green), nuclei (blue) in crypts cultured in the M1 coculture, M1-CM and M1 only culture models. C, (i) Histogram showing the average number of UEA-1 positive cells per crypt cultured in the M1 coculture, M1-CM and M1 only culture models and histograms showing the average number of UEA-1 positive cells per crypt region (C) (ii) base, (iii) mid and (iiv) top when cultured in the M1 coculture, M1-CM and M1 only culture models (*n* = 6, *P < .05 compared with control; $$$*P* < .001 compared with M1 coculture). Scale bar 20 μm. D, Histogram showing the average number of DCAMKL1 positive cells per crypt cultured in the M1 coculture, M1-CM and M1 only culture models and histograms showing the position of DCAMKL1+ cells per crypt region (ii) base (iii), mid (iv), and top in M1 coculture, M1-CM and M1 only culture models (*n* = 6, *P < .05 compared with control). Scale bars 20 μm.

We next sought to determine whether the reduction in UEA-1+ goblet cell and DCAMKL1+ tuft cell numbers in crypts cultured with M1 macrophages were induced via physical contact or a secreted factor/s and included an additional “M1 only” condition that had a Matrigel with crypts dotted next to a Matrigel with only M1 macrophages sharing the same medium to determine if M1 cells alone affected crypt differentiation (Supplementary Figure 3B experimental setup). Representative confocal images of control crypts, M1-CM, and M1 only macrophage conditions (Figure 5A) showed that UEA-1+ goblet cell (green) numbers were present at similar levels, but in the M1 coculture condition UEA1+ cell expression was less. Quantification of UEA+ cell numbers showed a significant decrease in the number of UEA-1+ cells in only the M1 coculture condition compared with control (Figure 5Ci), with a significant reduction in the mid crypt region (Figure 5Cii).

In a similar manner, we determined whether direct cell-cell contact between macrophages and colonic crypts was required to induce a reduction in DCAMKL1+ tuft cell number. Representative confocal images showed a reduction in DCAMKL1+ (green) tuft cells in M1 coculture, which were absent/rarely observed compared with control. The DCAMKL1+ cell expression in crypts of M1-CM and M1 only conditions were similar compared with control crypts (Figure 5B). Quantification of DKAMK1+ cells showed no change in M1-CM or M1 only compared with control, but M1 coculture showed a significant reduction in DCAMKL1+ tuft cell numbers (Figure 5Di), specifically at the base region of the colonic crypt (Figure 5 ii).

### Juxtracrine Contact Between M1 Macrophages and Colonic Crypts Is Required to Increase Lgr5+ Stem Cell Expression

**Figure 6.**
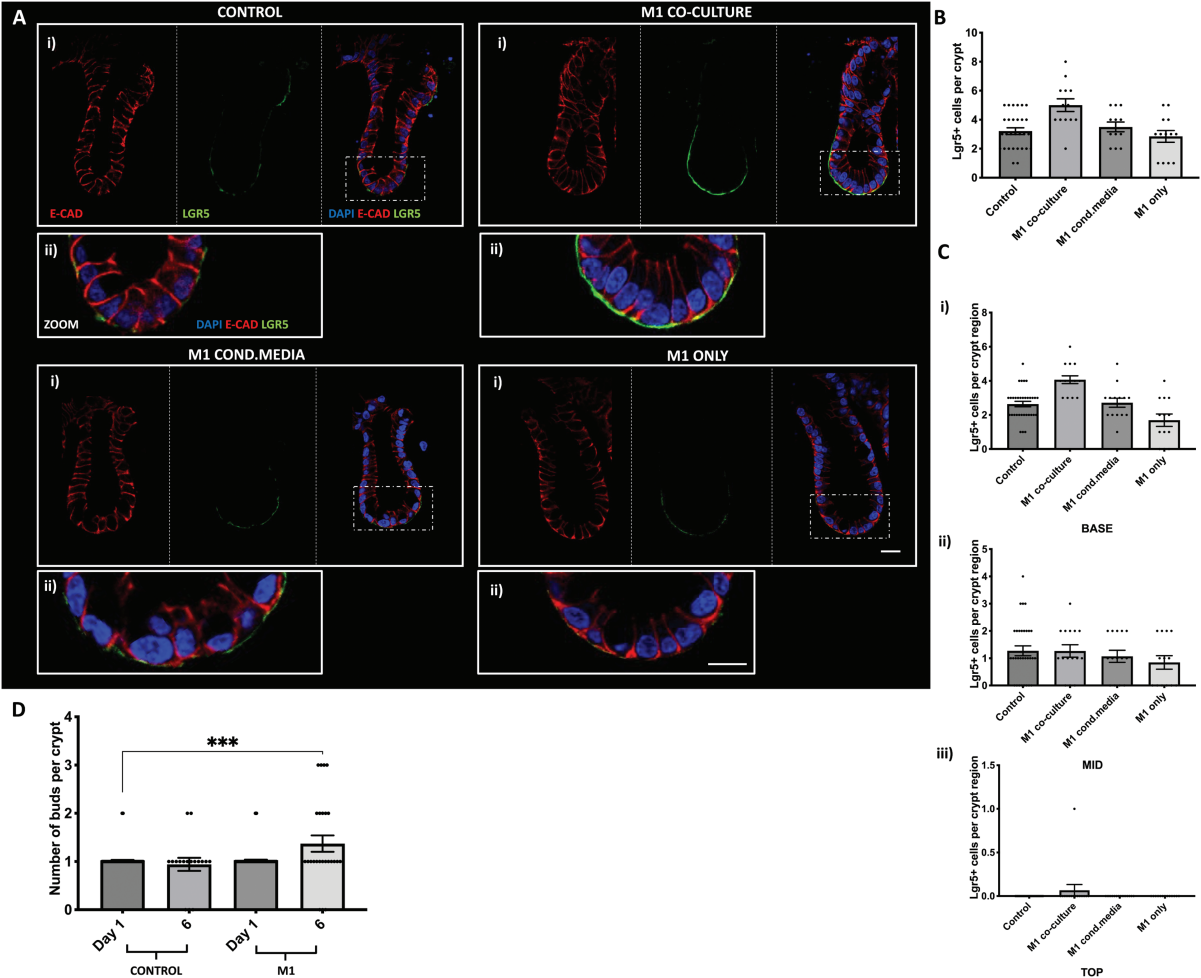
The M1 macrophages induce an increase in Lgr5+ cell expression via physical contact; not secretory factors in colonic crypts in vitro. A, (i) Representative confocal images showing basal Lgr5 expression (green), nuclei (blue) and E- cadherin (red) in crypts from M1 cocultures, M1-CM and M1 only culture models. A, (ii) Enlarged confocal images showing expression of Lgr5 (green) along the base of the crypt alongside white light or DAPI (blue), E-cadherin (red) when cultured in M1 coculture, M1-CM or M1- only models. B, Histogram showing the average number of Lgr5 positive cells per crypt cultured in M1 coculture, M1 and M1 only culture models. C, Histogram showing the position of Lgr5 + cells per crypt region (i) base, (ii) mid, and (iii) top when cultured in M1 coculture, M1-CM and M1 only models (*n* = 3, ***P < .001 compared with control; $*P* < .05, $$*P* < .01 M1 coculture compared with M1-CM; £££*P* < .001 M1 coculture compared with M1 only). D, Histogram showing the number of buds per crypt expressed on day 1 and 6 days in control crypts compared with M1-crypt coculture (*n* = 3; control vs M1 ***P < .0001). Scale bar at 20 µm.

Having previously established that M1 macrophages increase Lgr5+ stem cell expression in colonic crypts, we next determined whether macrophage-crypt contact or secreted factors are required to increase Lgr5 crypt stem cell expression. Representative confocal images of control crypts, M1 coculture, M1 only and M1-CM showed characteristic Lgr5+ (green) basal membrane labelling, with strongest labelling at the base, followed by mid and top regions of the crypt (Figure 6A). Quantification of the number of Lgr5+  + cells per crypt showed that M1-CM and M1 only conditions maintained Lgr5 cell numbers at similar levels to control crypts (Figure 6B). However, Lgr5+ cell numbers were significantly increased in the M1 coculture condition compared with control crypts (Figure 6B) and also a significant increase in crypt Lgr5+ stem numbers at the crypt base region compared with either the mid or top and compared with control (Figure 6Ci) The distribution of Lgr5+ cells along the crypt axis in all experimental conditions was similar with higher numbers at the base region, followed by the mid and top (Figure 6Ci-iii). Long-term (6 days) culture of M1 macrophages with colonic crypts (M1 coculture) resulted in a significant increase in colonic crypt budding compared with control crypts (Figure 6D).

### M1-Crypt Epithelial Contact Is Required to Increase Downstream Wnt Targets Proteins LEF1 and CyclinD1

**Figure 7.**
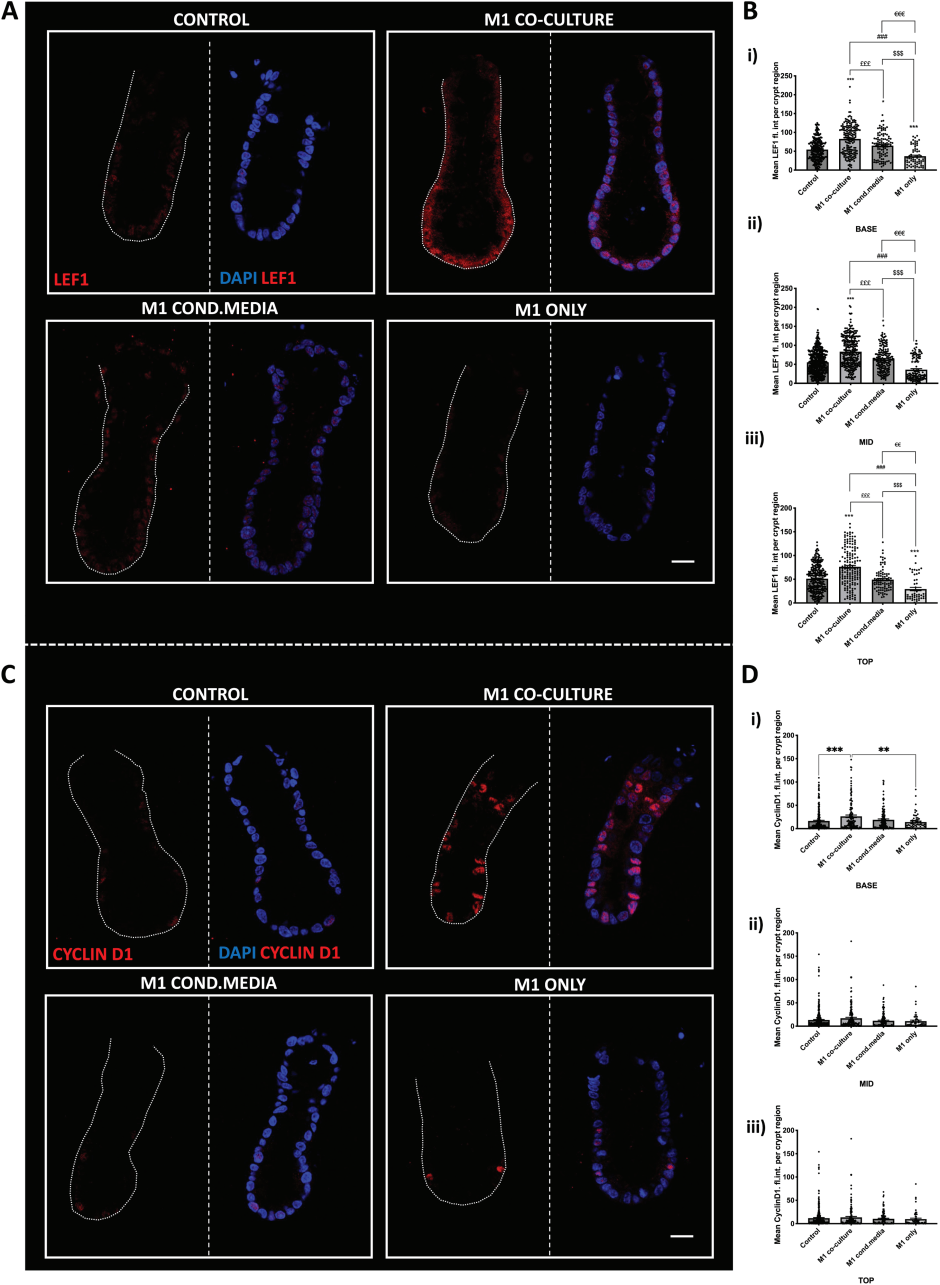
The M1 macrophages induce an increase in colonic LEF1 expression through physical contact and secretory factors, while colonic CyclinD1 expression is increased through physical contact but not secretory factors. A, Representative confocal images showing nuclear LEF1 expression (red), nuclei (blue) in crypts from M1 cocultures, M1-CM and M1 only culture models. B, Histogram showing the average fluorescence intensity of LEF1 within each crypt coculture/conditioned media experiment per crypt region (i) base (ii) mid (iii) top (*n* = 3, ***P < .001 compared with control; £££*P* < .001 M1 cocultured compared with M1-CM; $*P* < .05,$$*P* < .01,$$$*P* < .001 M1 coculture compared with M1 only). Scale bar at 20 µm. C, Representative confocal images showing nuclear Cyclin D1 expression (red), nuclei (blue) in crypts from M1 cocultures, M1-CM and M1 only culture models. D, Histogram showing the average fluorescence intensity of Cyclin D1 within each crypt coculture/conditioned media experiment per crypt region (i) base (ii) mid (iii) top (*n* = 3, compared with control. (*n* = 3, ***P < .001 compared with control; $$*P* < .01 M1 coculture compared with M1 only). Scale bar at 20 μm.

To determine whether physical cell-cell contact between macrophages and colonic crypts or coculture derived secretory factors differentially affect the Wnt target proteins, expression of CyclinD1 and LEF1 was further studied. Representative confocal images show LEF1 localization (red) in DAPI+ nuclei (blue) in all culture conditions, with increases in LEF labelling observed in the M1 coculture crypts and M1-CM to a lesser extent compared with control (Figure 7A). Semiquantitative analysis of the mean fluorescence intensity showed LEF1 expression was evenly distributed along the longitudinal crypt-axis in all conditions (Figure 7Bi-iii). The M1 coculture crypts and M1-CM crypts both showed a significant increase in LEF1 expression in the base and mid regions of the crypt compared with control; however, LEF1 expression in M1 coculture crypts was significantly higher at the base, mid, and top region of the crypts compared with M1-CM and control. The M1 only crypts caused a significant decrease in LEF1 labelling in all regions compared with M1 coculture, M1-CM, and control crypts.

Similarly, the expression of Cyclin D1 in each coculture condition was determined (Figure 7C). In control crypts, similar expression of CyclinD1 was found at the base, mid, and top of the crypt. In M1 coculture crypts, a significant increase in CyclinD1 expression was noted at the base of the crypt compared with control crypts; however, CyclinD1 expression was maintained at the mid and top region of crypts at levels similar to control. In M1-CM crypts and M1 only crypts, the mean fluorescence intensity of CyclinD1 expression remained unchanged at the base, mid, and top region of the crypt compared with control crypts. The mean CyclinD1 fluorescence intensity in M1-CM coculture crypts was significantly higher compared with M1 only crypts (Figure 7D).

## Discussion

Our study demonstrates that M1 and M2 bone marrow–differentiated macrophages differentially regulate colonic crypt renewal. Epithelial proliferation is increased by either subset, with M2 macrophages requiring crypt cell contact and M1 inducing growth through a secreted factor. The M2 macrophages maintain intestinal stem cell and differentiated cell numbers throughout the colonic crypt, while M1 macrophages reserve the unique ability to trigger an increase in Lgr5+ stem cells, as well as reduce UEA-1+ goblet cell and DCAMKL1+ tuft cell numbers in a juxtracrine-contact dependent manner. Furthermore, these M1-induced changes are accompanied by the upregulation of downstream Wnt-signaling targets LEF1 and CyclinD1.

Previous studies in which macrophages were ablated have observed a decrease in intestinal epithelial proliferation within in vivo injury models, yet it was not known whether macrophages are able to directly engage and affect the intestinal stem cell niche.^18,20^ We demonstrate that macrophages can directly increase epithelial proliferation in healthy colonic crypts through physical contact. Notably, known triggers of epithelial proliferation such as IL-6 and iNOS can be secreted by M1 macrophages proliferation.^25,26^ As contrasting signatures and cytokine profiles have been attributed to M1 and M2 macrophage phenotypes, we therefore postulate that these contribute to the differential epithelial proliferation we have observed in our study and will be the subject of future work.^10^

We demonstrate that juxtracrine interactions of the epithelium with M1 macrophages results in the reduction of goblet cells numbers, whereas M2 macrophages did not affect goblet cell numbers within the crypt-macrophage coculture model. The UEA-1+ mucus-producing goblet cells play a vital role in intestinal homeostasis, where ablation of goblet cells results in spontaneous colitis in mice, while goblet cell expression and its mucosal products are severely altered in patients with IBD.^13^ Interestingly, it has also been demonstrated that the ablation of macrophages in the small intestine results in increased goblet cell numbers, suggesting that goblet cell differentiation or fate can be regulated by intestinal macrophages. Other studies have shown a reduction in goblet cell expression in inflammatory bowel diseases, where the M1 macrophage phenotype is ubiquitously represented.^13,18,27^

The M1 macrophages also decreased the number of DCAMKL1+ tuft cells in colonic crypts through juxtracrine-contact, while crypt tuft cell numbers were maintained in the presence of M2 macrophages. The role of tuft cells within the intestinal epithelium is yet to be fully understood; however, colonic in vivo studies have shown that ablation of *Atoh1* a downstream Notch signaling transcription factor resulted in the depletion of DCMAKL1+ tuft cells in the colon.^28^ This raises the possibility that M1 macrophages may utilze the Notch signaling pathway to suppress Tuft cell differentiation. Interestingly, a decrease in tuft cell numbers was reported in patients with ulcerative colitis, an intestinal disease in which the activated M1 macrophages play an active role in disease progression.^14^ However, it is unclear whether macrophages can directly inhibit tuft cell expression or whether alterations in the Notch signaling cascade within the stem cell population resulted in the depletion of tuft cell differentiation within the colonic crypt. Therefore, further work must be undertaken to delineate the functional significance of tuft cells within the intestinal epithelium, which remains challenging due to the rare occurrence of this epithelial cell type. Within the secretory cell lineage, ChromograninA+ enteroendocrine cell numbers were maintained throughout the epithelium in our study. The differentiation of enteroendocrine cells from crypt progenitor cells requires the expression of *Atoh1* and *Neurogenin3*; and as studies have also shown that enteroendocrine cells are a highly conserved population within the intestinal epithelium,^29^ our findings suggest that it is unlikely that macrophages are able to influence the enteroendocrine cell fate.

Previous work from Sehgal and colleagues has shown that macrophages appear to be required for the maintenance of the intestinal stem cell niche, where the ablation of intestinal macrophages resulted in decreased Lgr5 mRNA expression in vivo.^18^ Here, we show that the M1 macrophage population significantly upregulate colonic Lgr5+ stem cell numbers in a juxtracrine-contact dependent manner. In the small intestine, Lgr5+ stem cell expression is commonly regulated via neighboring Paneth cells providing essential factors such as Wnt3a, EGF, TGF-α, and Notch ligand Dll4. However, the colonic epithelium lacks Paneth cell expression and likely relies on external stimuli and subepithelial cues for maintenance of the stem cell niche.^30^ It is therefore possible that a cell-cell contact through Notch or the short-range Wnt signaling pathway may be involved in inducing the changes within the stem cell population we have observed.^31,32^ Interestingly, earlier studies in human colonic epithelium have shown that Notch 1 was highly expressed in murine intestinal stem cells, while transcriptional profiling of bone marrow–derived M1 and M2 macrophages have demonstrated a significant increase in mRNA expression of the Notch ligands Delta-like ligand 1 and Jagged 1 in M1 macrophages when compared with nonactivated (M0) and M2 macrophages.^33^ The macrophages’ capacity to engage with the intestinal epithelium has been reported by numerous studies, especially in the colon, where most recently macrophages in the distal colon were shown to engage the epithelium through the formation of balloon-like protrusion used to limit the absorption of toxic fungal metabolites.^20,21,34^ As many of these findings are made in the large intestine, it is likely that the colonic epithelium is more receptive to macrophage-epithelial interactions compared with the small intestine, where the localization of Notch signaling receptors and ligands, specifically Jag-1, Dll-1, and Dll-4 in the colon, differs compared with the small intestine.^35^ The differential distribution of Notch ligands may allow macrophages with high Notch-signal receptor expression to engage and influence the intestinal stem cell niche within the colonic epithelium. However, further investigation is required to verify whether such a reciprocal interaction occurs in vivo.^36^

Recent work in the small intestine has suggested that Paneth cell-derived Wnt3a is directly transferred to Lgr5+ stem cells therewith regulating the intestinal stem cell niche.^31^ As our findings indicate that the increase in Lgr5+ stem cell expression is dependent on M1-macrophage contact, it is feasible that a similar mechanism is utilized. In support of this hypothesis, we have demonstrated that juxtracrine interactions between M1 macrophages and the epithelium results in an upregulation of LEF1 and Cyclin D1, both of which are key downstream canonical Wnt signaling targets. Wnt signaling is often aberrantly dysregulated in chronic inflammatory bowel diseases where M1-like macrophages are abundantly localized.^6^ Our findings may add to the notion that the M1 macrophage phenotype likely contributes to increased Wnt signaling activity, thereby exacerbating intestinal disease progression.^37^ Future work should first aim to untangle the complex upstream Wnt signaling cascades involved which led to the increased activation of LEF1 and Cyclin D1 observed in our reductionist M1-crypt coculture model, while succeeding studies should direct their focus towards understanding the role of pro-inflammatory macrophages on the epithelial stem cell niche using colitis mouse models.^38^

Our study offers a first insight into the overarching effects of macrophage subtypes on colonic crypt proliferation and differentiation. Fundamental differences between the effects of M1 and M2 macrophages are particularly apparent on crypt stem cell driven differentiation. Strikingly, the physical contact of macrophages with the crypt epithelium can have profound effects on renewal that are distinct for each of the macrophage subtypes in this study.

Very early work already suggested that epithelial turnover is increased in patients with ulcerative colitis (UC), while recent single-cell sequencing studies have shown that Lgr5 stem cell expression is highly enriched in healthy subjects compared with UC patients.^39,40^ Similarly, Lgr5 stem cell expression is also reduced in DSS (Dextran sodium sulfate)-induced colitis mouse models, altogether suggesting that stem cell dysregulation is likely a key marker of IBD pathology.^41^ In this study, we have highlighted the significance of direct macrophage-epithelial contact and hint at the potential role of macrophages as a regulator of Lgr5 stem cell renewal. Future studies should endeavor to closely examine the signaling mechanism involved such as the Notch or Wnt signaling pathway to understand the dialogue between these 2 cell types, which may allow us to exploit the mechanism to restore epithelial repair in patients with IBD. Further work should also aim to define the exact macrophage phenotype required to trigger stem cell expansions, which may allow clinicians to utilize such markers as a prognostic tool and could also be used to score the patient severity of the disease going forward.

## Supplementary Data

Supplementary data is available at *Inflammatory Bowel Diseases* online.

izad270_suppl_Supplementary_Material
